# Variation in Leg Tissue Dielectric Constant Values of Healthy Young Adult Females With and Without Compression Bandaging

**DOI:** 10.7759/cureus.38647

**Published:** 2023-05-06

**Authors:** Suzie Ehmann, Harvey N Mayrovitz

**Affiliations:** 1 Physical Therapy, Nova Southeastern University, Fort Lauderdale, USA; 2 Medical Education, Nova Southeastern University Dr. Kiran C. Patel College of Allopathic Medicine, Davie, USA

**Keywords:** leg circumferences, interface pressure, leg edema, edema, lymphedema, compression, tissue dielectric constant, tdc

## Abstract

Background

The clinical efficacy of a compression application has been often limited to the assessment of the change in limb volume, change in clinical symptoms (i.e., wound size, pain, range of motion, incidence of cellulitis), or vascular hemodynamics of the whole limb. Assessing compression-related biophysical changes of a localized area, such as around a wound, or in an area outside of an extremity cannot be objectively assessed by these measurements. Tissue dielectric constant (TDC) values, which provide a measure of the local tissue water (LTW) content, offer an alternative method to document variation in the LTW content of the skin in a specific location. The goals of the present research were (1) to characterize TDC values, expressed as percentage tissue water, from multiple areas along the medial aspect of the lower leg of healthy volunteers and (2) to explore the potential utilization of the TDC values to assess change in tissue water content in a localized area following compression applications.

Methods

TDC was measured at 10, 20, 30, and 40 cm proximal to the medial malleolus on the medial aspect of the right leg of 18 young adult healthy women with an age range of 18-23 years and a body mass index of 18.7 to 30.7kg/m^2.^. TDC was measured at baseline and after 10 minutes of exercise with compression in place on three separate days during which three different compression applications were assessed: a longitudinal elastic stockinette, a two-layer cohesive compression kit, and a combination of the two. Leg circumferences and compression-related interface pressures were also measured.

Results

Test-Retest Reliability of circumferential measurements and TDC values evaluated using Intraclass correlation coefficient (ICC _3,1_) revealed excellent and moderate-to-good reliability, respectively. Analysis of TDC values along the length of the limb using Friedman’s test, revealed a small but statistically significant overall difference among baseline TDC values attributable to a smaller value at 40 cm. The largest difference in cumulative average was 7.7% which occurred between 20 and 40 cm, with all other differences between locations less than 1%. No significant differences between the compression applications were observed.

Conclusion

The present findings demonstrate the utility of TDC measurements as a modality to assess compression-related changes in the legs of healthy women as a foundation for their potential use in assessing outcomes of compression treatments for persons with lower extremity edema or lymphedema. The absence of a significant change in TDC values in these healthy non-edematous conditions and the demonstrated reliability of the TDC measurements on three different days provides further support for the utility of such applications of TDC measurements. The extension to patients with lower extremity edema or lymphedema needs to be evaluated.

## Introduction

Compression is an established therapeutic tool utilized for the management of edema of multiple etiologies and in the management of venous leg ulcers (VLU) [1-10]. Therapeutic benefits of compression documented in the literature include improved circulation, integumentary restoration, and improved clinical symptoms [2,3,11,12]. Clinical efficacy of a compression application in the literature has been often limited to the assessment of the change in limb volume, change in clinical symptoms (i.e., wound size, pain, range of motion, incidence of cellulitis), or vascular hemodynamics of the whole limb [1,13-20]. However, assessing compression-related biophysical changes in a localized area, such as around a wound, or in an area outside of an extremity cannot be assessed by these methods. Tissue dielectric constant (TDC) values, which provide a measure of the local tissue water content (LTWC) of a specific area, offer an alternative method to document biophysical variations of the skin in a specific location [21]. Such localized assessments can provide insight into the effects of a treatment intervention at localized areas such as those associated with a compression bandage or compression application of various types of specific interest. To date, such TDC measurements at multiple target sites in combination with compression on the lower leg has not been reported. The goals of the present research were (1) to characterize TDC values from multiple areas along the medial aspect of the lower leg of healthy volunteers and (2) to explore the potential utilization of the TDC values to assess change in tissue water content in a localized area following the application compression applications.

## Materials and methods

Subjects

The purpose of the study was to gain previously unavailable information and as such might be viewed as a pilot study. The use of females was a convenience of availability as well as numbers (n=18). Subjects studied were healthy volunteers, 18 years of age or greater, recruited via electronic flyer from the Clemson University student body and employees in the engineering program. To be included, subjects needed to assure they had the ability to ambulate for 10 continuous minutes on a treadmill (TM) at a minimum speed of 2.0mph. Subjects were ineligible from participating if they had any of the following: visible signs of chronic venous disease (CVD) such as hemosiderin staining or visible varicosities, history of any trauma to leg, ankle, or foot within the last six weeks, history of idiopathic swelling or vascular malformations, allergies to the compression material. Interested participants deemed eligible to participate signed an informed consent approved by Clemson University Institutional Review Board (IRB 2022-0475). The subject’s age (mean ± SD) was 19.3 ± 1.2 years with a range of 18 to 23 years. Body mass index (kg/m^2^) was 22.4 ± 3.3 with a range of 18.7 to 30.7.

Compression materials

The compression used in the management of edema and CVD entails the application of a textile around a limb which creates pressure. In the outpatient wound care setting, disposable boxed compression sets or tubular elastic stockinets are the mainstays of compression treatment. These compression applications are traditionally classified by the number of layers (2-, 3-, 4-layer), textile properties (i.e., short stretch, elastic, cohesive), and/or expected compression pressure dosage (mmHg). For the present study, the compression applications chosen were Co-Flex® TLC (Milliken Healthcare, Spartanburg, SC, USA), EdemaWear® (Compression Dynamics, Omaha, Nebraska, USA), and a combination of the two compression products simultaneously applied. 

Co-Flex® TLC system is classified as a two-layer cohesive box set. The first layer is referenced as a comfort foam layer with no stated compressive qualities. This layer is applied in a spiral fashion around the foot with one figure of eight wraps around the heel/ankle, and then continued spiral wrapping up the leg with each contiguous wrap with 50% overlap with the preceding, with just enough tension to conform to the shape of the leg and ending just below the knee. The second layer is denoted as the compression layer and is a non-latex short stretch cohesive compression bandage containing visual indicators to facilitate proper tension with the application. Directions for application are similar to the first layer, applying in a spiral fashion around the foot, two figure eights around the heel/ankle, completely covering the heel, and then continuing the spiral wrapping up the leg ending just below the knee. This product is disposable, designed for single use only. CoFlex® TLC is indicated for the management of venous leg ulcers (VLU) and related conditions, for those with an ankle brachial pressure index (ABPI) ≥ 0.8. There is only one size of CoFlex® TLC. The manufacturer’s stated expected dosage is 30-40mmHg.

EdemaWear® is a tubular, circular knit elastic stockinet fabricated with elastic, vertically oriented fuzzy wales, with the compression provided by the elastic, horizontally oriented Lycra® spandex yarns knitted in between. The direction for the application of EdemaWear® is similar to the application of a traditional compression sock. The stockinet is applied from the foot to the popliteal crease, over the affected area, with the EdemaWear® in direct contact with the skin. This product is reusable and can be laundered and cut to fit. Sizing is based on leg circumference. A size small was used for the present study. EdemaWear® is indicated for edema management of various etiologies (lymphedema, CVD, congestive heart failure, etc.), and optimizing wound healing regardless of ABPI. The stated dosage is 8-12mmHg.

Measurements

TDC measurement is a non-invasive, convenient, reliable, and accurate method to assess LTWC [22-25]. Using an open-ended coaxial probe, a low-power 300MHz signal is transmitted into the skin when the measuring sensor of the device is placed in contact with the skin. A portion of the transmitted energy is reflected to the probe and a processor determines the TDC value [26]. The TDC value is a dimensionless physical quantity that is directly related to LTWC [26]. The TDC value that is calculated includes contributions of both free and bound water in the tissue [27,28]. TDC has been used to assess lower limb edema, lymphedema, and both post-operative and post-treatment-related changes in localized tissue water [24,29-44].

The bulk of the clinical research published has been devoted to the quantitative assessment of localized tissue edema related to lymphatic impairment following breast cancer treatment. This research is focused on the upper extremity, the breast, and/or trunk [24,34-36,44-51]. There are few studies evaluating the utilization of TDC to quantitatively assess the clinical presentation of lower extremity edema and/or lymphedema [23,30,31,43,52-55].

The validity and reliability of the TDC as a measure of tissue water content, specifically for the lower leg has been demonstrated in vitro and in vivo [21,23,25,26,30,56]. Reliability of the TDC measurement has been documented to vary by specific location, device utilized, number of measurements performed, and specific patient population (healthy vs. diseased) [21-23,27-30,37,49,54,56-59]. Although research on the upper extremity is more comprehensive demonstrating a larger variety of assessment locations and devices utilized, a focused review of published studies examining the assessment of TDC in the lower extremity reveals the measurement points studied were localized to a singular spatial location (i.e., calf, dorsum of foot, thigh) limiting the clinical utilization of this data for the current project. Knowledge of the spatial variability in healthy subjects in a more focused area, as an example the lower leg below the knee which is commonly covered by a compression bandage or garment, can serve as a basis for further examination of the utilization of TDC to assess the impact of a treatment intervention in those individuals with pathology.

The TDC device utilized was the MoistureMeterD compact (MMDC) (Delfin Technologies Ltd., Finland). It is a light, handheld device, which has a built-in pressure sensor aiding consistency of contact pressure and a display window providing LTWC in percent (0%-100%) [57]. The device has an effective measurement depth of 2.0-2.5mm [54,57].

Compression pressure measurements

Prescription and efficacy of a compression application have traditionally centered on the pressure produced at the interface of compression textile and the skin. Measurement of this direct IP under a compression application can be accomplished by a variety of devices as detailed in Table 1 [60]. Although pneumatic-based systems are the most common type of measurement system used in measuring compression in clinical practice, they only provide a point pressure measurement. Pneumatic-based systems are not able to objectively document the pressure distribution across the surface of the limb over which a compression application has been applied. Flexible piezoelectric sensors can objectively document not only the average point pressures but also the peak pressures between and pressure distribution across the entire surface of the sensor. As one of the aims of the study at large was to assess the in vivo IP in both the horizontal and vertical distribution under different compression conditions, the TEKscan (Norwood, MA, USA) I-Scan® piezoresistance sensor was chosen.

**Table 1:** Types of sensors used to measure IP under compression applications.

Sensor Type	Mechanism of Measure	Example
Pneumatic	Bladder-like pocket is filled with air and the measured pressure is transmitted to a pressure transducer and displayed. May also have an electrical component where the pressure is converted into an electrical signal using a pressure transducer.	PicoPress® (Microlab, Padua, Italy) Kikuhime®(Meditrade, Doro, Denmark) SIGaT-test® (Ganzoni-Sigvaris, St Gallen, Switzerland) Juzo Pressure Monitor®
Piezoelectric	Mechanical stress produced by the application of a force on the sensor creates an electric charge which is transmitted via electrical lead.	Smart Sleeve Pressure Monitor® Carolon Company, Rural Hall, North Carolina.
Piezoresistive	Thin and flexible. Consists of multiple layers including pressure-sensitive or piezoresistive film with integrated electrical resistance properties which vary with applied pressures enabling calculation of force per unit area	I-scan®(TEKscan) (Norwood, Massachusetts, USA) Flexiforce®(TEKscan) (Norwood, Massachusetts, USA)
Capacitive	Consists of electrical elements that store energy which changes in response to pressure; this change in energy is translated into electrical capacitance to give a measurement of pressure	Pliance X System (Germnay-Novel Electronics, Munich, Germany)

The TEKscan I-scan® sensor is a thin film piezoresistive tactile pressure sensor with the capacity to capture pressure distribution of different compression applications in both the vertical and horizontal dimensions as was previously demonstrated in vitro [61]. The TEKscan I-scan® 6300 has a rectangular shape providing a sensing area along the vertical length of the limb to capture continuous vertical and horizontal pressure distributions along the contour of the limb. The sensor specifications are detailed in Table 2.

**Table 2:** Interphase sensor specifics. TEKscan I-scan® Sensor 6,300 has high spatial resolution with more than 25 sensels/cm^2^. The sensor was placed on the medial aspect of the right leg prior to the application of compression.

Sensor Type	Matrix Height	Matrix Width	Resolution
TEKscan I-scan® Sensor 6300	33.5mm	264.2mm	2288 sensels (25.8 sensels/cm^2^)

The study protocol and outcome measurement procedures 

**Figure 1:**
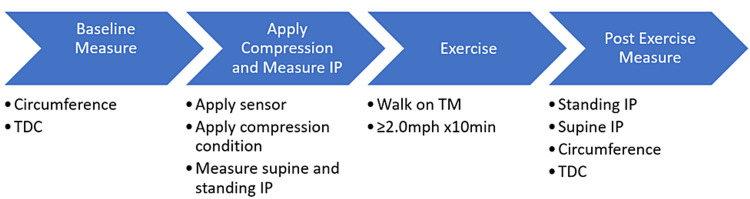
Sequential procedures. Procedures were repeated on each subsequent visit (total of three visits, on three different days, for each subject). The number of complete observations for each compression condition was 40.  TDC, tissue dielectric constant; IP, interface pressure; TM, treadmill.

The data presented in this paper represent one outcome measure that was collected as part of a multi-phase research project to comparatively assess compression applications in a healthy population. The study required subjects to attend the lab on three different days, at approximately the same time of day for all sessions. A different compression condition, assigned randomly via a balanced Latin square generator, was applied each day. Upon arrival at the laboratory, subjects rested in a seated position with their feet on the floor for five minutes. The study protocol after the five-minute rest break is illustrated in Figure 1.

**Figure 2:**
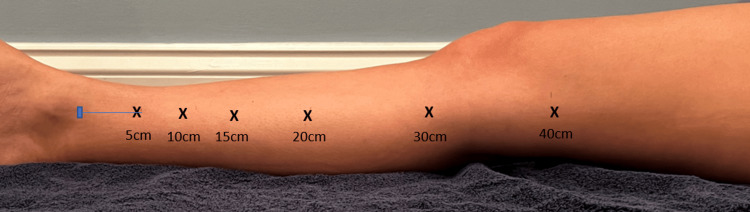
Measuring locations. Six measurement locations were identified on the medal aspect of the right leg. Circumferential measurements were made at all six and LTWC was assessed at 10-, 20-, 30-, 40cm. LTWC, local tissue water content.

After five minutes of seated rest, the subject was asked to assume a semi-reclined position on a folding massage table with the right lower extremity fully extended and the left lower extremity positioned off the table on the floor or a stool to allow full access to the medial aspect of the limb. The superior aspect of the medial malleoli was palpated and from this anatomical landmark, a plastic tape measure was used to measure, and an indelible marker was used to mark 5-, 10-, 15-, 20-, 30-, and 40-cm proximally as depicted in Figure 2.

Circumferential measurements were made once at each of the measurement locations. TDC was measured once at the 10-, 20-, 30-, and 40-cm mark starting at the distal location. The starting point at the 10cm mark was chosen because it was similar to other researchers’ measurements of the lower limb. Next, the piezoelectric sensor was sprayed with skin adhesive and placed on the medial aspect of the limb. After the sensor was in place, a thin non-compressive nylon stocking was placed over the leg and that day’s designated compression condition was applied.

The subject then walked on a TM for 10 minutes at a self-selected speed they deemed a comfortable pace they could maintain for 10 minutes, but at a minimum speed of 2.0 mph. After walking on the TM for 10 minutes the subject returned to the massage table and assumed the same position with the right lower extremity fully extended on the table and the left leg resting on the floor. After the compression application and the sensor was removed, three additional TDC measurement sets were taken. The first set was immediately after removal of the compression wrap, the second and third sets were started two and five minutes after removal of the compression product. Each subject returned for two additional sessions, at relatively the same time of day. The mean time between treatment visits was 36 hours (SD 11.5; range 18-72 hours).

Data analysis

The data were analyzed using IBM SPSS Statistics version 28 (IBM, Armonk, NY, USA). A complete data set was captured for all female subjects (n=18), on all three days. For the normality of variables, normality (Kolmogorov-Smirnov and Shapiro-Wilk) tests were conducted. Test-retest and intrarater reliability was analyzed using the agreement between the measurements. The agreement between the measurements was analyzed by the ICC_3,1_ single rating, absolute agreement, a two-way random-effect model with one rater, across 18 subjects. The two-way mixed-effect model was used to test intrarater reliability with multiple measurements from one rater as it is not reasonable to generalize one rater’s scores to a larger population of raters [62]. Furthermore, as repeated measures cannot be regarded as randomized samples, this model was used to assess the test-retest reliability of the baseline circumference and TDC measurements taken on three different days. The absolute agreement definition was chosen for both test-retest and intrarater reliability studies because measurements would be meaningless if there is no agreement between repeated measurements [62]. The strength of the ICC values was interpreted according to Portney and Watkins, where values below 0.5 represent poor reliability, values of 0.50-0.75 represent moderate reliability, values between 0.75 and 0.9 indicate good reliability, and values greater than 0.90 represent excellent reliability [63]. The non-parametric Friedman repeated measures test was used to determine if there was a significant change over time (pre-exercise, immediately after exercise, and two-, and five-minute post-exercise). A non-parametric Wilcoxon test was performed to test the significance of pairwise differences between measurement points. An overall 5% type I error level was used to infer statistical significance.

## Results

Baseline circumferences

The average circumference of the three sessions, at baseline and five minutes after compression removal, is detailed for each measurement location in Table 3. For both, excellent reliability was observed. The confidence interval (CI) was narrow for all measurement locations. There was no significant change in circumference post-intervention.

**Table 3:** Leg circumferential measurements. Baseline and post-intervention circumferential measurements at six measuring points on the lower limb of healthy women (N=18). Data in the table are presented as mean ± SD (Range). Test-Retest Reliability of circumferential measurement presented as 95% CI for ICC. ICC, intraclass correlation coefficient; CI, confidence interval.

	Baseline		Post-Intervention	
Measurement Location	Circumference (cm)	95% CI for ICC_(3,1)_	Circumference (cm)	95% CI for ICC_(3,1)_
5cm	22.7 ± 1.5 (20.7 - 25.2)	0.967 (0.928 - 0.987)	22.8 ± 1.4 (20.6 - 25)	0.938 (0.865 - 0.975)
10cm	26.9 ± 1.9 (23.7 - 30.5)	0.970 (0.934 - 0.988)	26.9 ± 1.9 (23.7 - 30.4)	0.980 (0.955 - 0.992)
15cm	30.8 ± 2.6 (27.2 - 36.3)	0.982 (0.961 - 0.993)	31.6 ± 2.4 (27.0 - 36.4)	0.986 (0.968 - 0.994)
20cm	35.3 ± 2.0 (31.3 - 39.6)	0.963 (0.919 - 0.985)	35.2 ± 2.1 (31.2 - 39.4)	0.992 (0.983 - 0.997)
30cm	33.9 ± 2.2 (29.4 - 37.3)	0.991 (0.979 - 0.996)	33.8 ± 2.2 (29.5 - 37.3)	0.994 (0.986 - 0.997)
40cm	37.7 ± 3.0 (33.4 - 43.8)	0.992 (0.983 - 0.997)	37.8 ± 3.1 (33.5 - 44.6)	0.994 (0.988 - 0.998)

Baseline IPs

Baseline IP measured in supine (IP_Supine_) and in standing (IP_Stand_) are detailed in Table 4. Among subjects, there was a wide range of IP values measured at the ankle (4-75mmHg) and the calf (3-80mmHg). As shown in the table, the highest group average IP was observed with the combination of EdemaWear® and CoFlex TLC at all time points and both locations.

**Table 4:** Interface Pressures. IP measured at ankle and calf immediately after application of each compression condition (Baseline) and post 10 minutes of exercise. Data in the table are presented as mean ± SD and (Range). SD, standard deviation; IP, interface pressure.

	Baseline IP (mmHg)		Post Exercise IP (mmHg)	
	Supine	Standing	Supine	Standing
EdemaWear®				
Ankle	10.7 ± 4.2 (4 - 23)	13.8 ± 5.2 (5 - 30)	12.5 ± 4.6 (4 - 26)	14.9 ± 5.2 (5 - 30)
Calf	10.7 ± 3.6 (3 - 23)	12.6 ± 4.7 (5 - 31)	11.6 ± 4.8 (5 - 26)	14.6 ± 5.5 (5 - 32)
CoFlex® TLC				
Ankle	27.9 ± 8.7 (8 - 58)	31.6 ± 9.9 (9 - 57)	25.4 ± 9.3 (8 - 56)	35.25 ± 11.2 (11 - 68)
Calf	24.7 ± 9.3 (9 - 48)	26.6 ± 10.4 (10 - 55)	17.4 ± 7.8 (6 - 36)	28.8 ± 10.7 (11 - 54)
EdemaWear® + CoFlex® TLC				
Ankle	37.5 ± 9.8 (9 - 56)	42.6 ± 11.9 (11 - 69)	27.0 ± 8.9 (9 – 55)	40.0 ± 12.9 (12 - 75)
Calf	35.2 ± 11.9 (12 - 66)	38.3 ± 13.4 (13 - 67)	22.4 ± 10.1 (10 - 60)	37 ± 13.8 (14 - 80)

Baseline TDC values

Baseline TDC values, expressed as percentage water, measured at 10, 20, 30, and 40 cm proximal to the medial malleolus on each of the three sessions are detailed in Table 5. The reliability of a single baseline measurement across the three sessions, characterized by the ICC value, was moderate at the 10cm location and good at the others. Analysis of the values along the length of the limb using Friedman’s test revealed a significant overall difference among baseline TDC values attributable to the smaller value at 40 cm. The largest difference in cumulative average was 7.7% which occurred between 20 and 40 cm, with all other differences between sites less than 1%.

**Table 5:** Baseline tissue dielectric constant values. Cumulative average and detailed TDC values expressed as percentage water for each treatment session, at four locations on the medial aspect of the right lower leg of healthy women (N=18). Test-Retest Reliability of TDC (%) is presented as 95% CI for ICC. TDC, Tissue Dielectric Constant; ICC, intraclass correlation coefficient; CI, confidence interval.

Location	Cumulative Average	Session 1	Session 2	Session 3	95% CI for ICC_(3,1)_
10cm	43.5 ± 3.4	43.9 ± 3.8 (39.2 – 51.9)	43.5 ± 3.1 (38.2 – 51.4)	43.5 ± 3.8 (34.3 – 52.1)	0.730 (0.409 - 0.893)
20cm	43.9 ± 2.9	44.2 ± 3.9 (38.4 – 52.1)	44.1 ± 2.9 (39.3 – 51.4)	44.2 ± 4.5 (34.3 – 52.2)	.836 (0.636 - 0.934)
30cm	43.6 ± 2.7	42.4 ± 2.9 (34.3 – 45.9)	42.6 ± 2.5 (38.2 – 46.2)	42.7 ± 3.7 (34.3 – 51.3)	.848 (0.664 - 0.939)
40cm	40.5 ± 2.8	40.9 ± 3.1 (34.2 – 46.2)	41.4 ± 2.7 (38.1 – 46.0)	40.8 ± 3.6 (34.3 – 46.0)	.880 (.738 - .951)

TDC differences among compression conditions

Table 6 summarizes the TDC data for each compression condition at each measured leg site. Post-hoc analysis with Wilcoxon signed rank tests was conducted to further analyze the significance of pairwise differences between measurement points relative to compression condition, spatial location on leg, and time. Statistical differences were observed between TDC measurements taken from the area of the leg covered by the compression application (10-, 20-, 30cm) and a measurement taken just proximal to the compression application (40cm). Analysis of the data for the individual compression conditions demonstrated there was a significant difference in TDC measurements noted at positions 30cm and 40cm. At the 30cm mark, the smallest significant change was noted with EdemaWear, z=2.3, p=0.022. The largest significant change was noted with Coflex® TLC and the combined application of the EdemaWear and the Coflex® TLC, z=-2.22, p=0.26 and z=-2.18, p=0.29, respectively. At the 40cm measurement point, a significant change was noted only with the combined application, z=-1.97, p=0.049. Analysis of the cumulative change in TDC values demonstrated there was an observed difference between compression conditions at only the most distal measurement point (10cm), c2=9.49, p=0.009. Post hoc analysis with the Wilcoxon sign rank test demonstrated the difference was noted between Coflex® TLC and EdemaWear only, z=-2.483, p=0.013. TDC percent change varied greatly by location and condition, with the greatest mean reduction observed under a compression application observed at measurement point P10 with Coflex® TLC (-1.04% ± 6.32).

**Table 6:** Effect of intervention on tissue dielectric constant. Tissue dielectric constant expressed as percent water measured on the medial aspect of the right lower limb of healthy women (N=18). Data are presented as mean ± SD (range) for each test condition. Cumulative average calculated from measurements made immediately after compression removal, and again at two and five minutes. Wilcoxson Sign Ranks test for the post exercise change with significance level (p) is denoted. SD, standard deviation.

	EdemaWear®		CoFlex® TLC		EdemaWear® + CoFlex® TLC	
Location	Baseline	Post Exercise	Baseline	Post Exercise	Baseline	Post Exercise
10cm	44 ± 3.8 (39-52)	44.6 ± 4.1 p=0.102	43.5 ± 3.1 (38-51)	42.9 ± 2.8 p=0.408	43.5 ± 3.8 (34-52)	43.1 ± 4.2 p=0.256
20cm	44.2 ± 4.0 (38-52)	44.1 ± 3.4 p= 0.811	44.1 ± 3.0 (39-51)	44.0 ± 2.4 p=0.828	44.2 ± 4.5 (34–52)	43.8 ± 4.7 p=0.538
30cm	42.4 ± 3.0 (34-46)	43.2 ± 3.0 p=0.022	42.6 ± 2.5 (38-46)	43.5 ± 2.3 p=0.026	42.7 ± 3.7 (34–51)	44.2 ± 4.2 p=0.029
40cm	40.9 ± 3.1 (34-46)	40.5 ± 3.2 p=0.223	41.4 ± 2.7 (38-46)	40.8 ± 2.5 p=0.062	40.8 ± 3.6 (34-51)	40.3 ± 3.9 p=0.049

## Discussion

Measuring the impact of a compression application, beyond segmental changes in circumferential measurements, has the potential to provide insight into the biophysical impact of a specific compression textile locally. TDC, a quantifiable measure of LTWC, is an adjunctive measure that can be used to monitor the impact of a compression application in a localized area over which compression is applied, affording greater insight into local effects [22]. Previous research has demonstrated that there is substantial variation in TDC values that occurs among anatomical sites on the lower extremity and clinical devices [21,23,27,30]. Explanations for the observed variations include differences in tissue composition at each of the assessed locations and the device utilized [27]. Subcutis and fat are known to have relatively lower water content, while dermis and connective tissue (including tendons, blood vessels, bones, and nerves) have relatively higher water content [21,64]. Similarly, in a device that is measuring a shallower depth measurement, where there is less subcutaneous fat, the TDC will be higher relative to values measured by a device assessing at a greater depth [21].

Previous researchers utilizing TDC to assess the lower extremity have used segmental measuring points to be representative of the whole leg [21,23,29-31,43,52]. As an example, Jonsson et al. looked at 14 different measurement points on the lower leg. Of these, only six assessed the lower extremity below the knee, at only two spatial locations circumferentially around the leg [23, 30]. The author did not comment on the significance of the differences between these measurement points. Mayrovitz [55], and Birkballe et al. [29] used three measuring points each: 1) the dorsum of the foot and medial and lateral aspect of the lower leg at the same vertical height; 2) the dorsum of the foot, medial ankle and a singular point on the lateral lower leg, respectively. Mayrovitz utilized a ratio of calf/forearm TDC ratio exceeding 1.35 as a clinical measure suggestive of the presence of lymphedema [55]. Birkballe et al. utilized the previously identified measurement points to demonstrate discriminative value between clinical presentation of treated and untreated lymphedema, and lipedema [29]. Utilizing these landmarks, the researchers observed a significant difference between the groups assessed but not within the groups themselves, observing that the foot measurements were significantly higher than the ankle and lower leg measurements in the treated lymphedema, lipedema, and healthy controls [55]. Whereas the foot TDC values for the untreated lymphedema group were significantly below the lower leg values [55].

Utilization of a single measurement point to be representative of a larger segmental area can obscure or minimize the effect of a focal treatment, such as a compression application applied only to a portion of the limb or the cumulative effect of a compression application or a topical dressing applied to a wound under a compression application. As was observed in this research, there were small, but significant differences in baseline measures of LTWC spatially along the medial aspect of the leg of the healthy volunteers that remained following the test conditions. This variation has been previously explained by researchers relative to the different anatomical regions and tissue composition being assessed. However, if only a single measurement point in the lower leg was utilized, the change in TDC following the test conditions would have been unnoticed as it was not captured with circumferential measurements that were virtually unchanged. Although the change in TDC following the test conditions in this research was small, it was not captured with circumferential measurements.

Comparison of observed baseline measurements and outcomes with published research is hindered due to dissimilar methodology, use of different measurement points, and different devices. Comparison requires conversion of the data collected (%) to a TDC. The output of the MMDC device used in this research is referred to as LTW, which is derived from the actual TDC measurement according to the formula LTW(%)=100% x (TDC-1)/77 [28]. This comparison is further complicated by the difference in the depth of measurement with the device used in this research, MMDC (measurement depth 2-2.5mm); whereas the effective measurement depth of the MoistureMeterD is 2.5mm. The inability to direct comparative analysis of the findings of this research to previous research highlights the importance of referencing a more precise location and using the same device when using TDC measurements in patients to detect, track or evaluate treatment effects in the lower leg.

The inability of the TDC measurements to discriminate between the compression applications in the healthy sample population was not unexpected as the subjects had no baseline edema that would have been impacted by the compression applications regardless of the compression dosage (mmHg).

Limitations of this research include the small size and relative uniformity of our study population with respect to age, gender, and BMI distribution poses a limitation on direct comparisons to the same measure in male, obese, or elderly populations. Since the investigation was performed on healthy subjects free of edema, the extent to which these results apply to patients with edematous presentations is not known and still needs to be explored.

## Conclusions

The present findings demonstrate the utility of TDC measurements as a modality to assess compression-related changes in the legs of healthy women as a foundation for their potential use in assessing outcomes of compression treatments for persons with lower extremity edema or lymphedema. The absence of a significant change in TDC values in these healthy non-edematous conditions and the demonstrated reliability of the TDC measurements on three different days provides further support for the utility of such applications of TDC measurements. The extension to patients with lower extremity edema or lymphedema needs to be evaluated.
